# Isolation of aerobic bacteria from surgical site infections following orthopaedic operations in cats and dogs

**DOI:** 10.17221/26/2024-VETMED

**Published:** 2024-07-29

**Authors:** Ali Gulaydin, Ozgul Gulaydin, Mustafa Baris Akgul

**Affiliations:** ^1^Department of Surgery, Faculty of Veterinary Medicine, Siirt University, Siirt, Turkiye; ^2^Department of Microbiology, Faculty of Veterinary Medicine, Siirt University, Siirt, Turkiye

**Keywords:** antimicrobial resistance, cat, dog, *Staphylococcus pseudintermedius*, surgery

## Abstract

Surgical site infections (SSIs) cause significant complications in the postoperative period in veterinary surgeries. Determining the aetiology of infections is crucial for increasing treatment success rates. This study aims to assess treatment processes by identifying the bacterial agents responsible for infections occurring in cats and dogs after orthopaedic operations and to determine the antimicrobial resistance profiles of these agents. Strains isolated from SSIs were retrospectively analysed in patients brought to the Surgical Clinic of Siirt University Animal Health Application and Research Hospital between 2021 and 2023. The isolates were identified using MALDI-TOF MS. The disc diffusion method was applied to determine the antimicrobial susceptibility of the isolates. A high isolation rate was detected in methicillin-resistant *Staphylococcus pseudintermedius* and extended-spectrum beta-lactamase (ESBL) producing *Enterobacteriaceae*. According to the antimicrobial susceptibility results, cephalosporin treatment was continued in only one of the cases in which prophylactic treatment with cephalosporin group antibiotics was applied. Consequently, this study revealed that preoperative prophylactic antibiotic administration may not be sufficient in preventing surgical site infections. Diagnosis of aetiological agents and evaluation of antimicrobial susceptibility are essential in preventing surgical site infections and determining effective treatment options.

Surgical site infections (SSIs), commonly defined as wound infections that develop within 30 days postoperatively, also include infections occurring within a year after implant applications (Weese 2008). The patient’s physiological and genetic characteristics, environmental factors, and other factors during preoperative, postoperative, and intraoperative periods contribute to the development of this infection (Devriendt et al. 2023).

Prophylactic antimicrobial therapy aims to prevent SSIs and reach an antimicrobial level that prevents bacterial adhesion in the tissues and reduces the potential risk of postoperative infection (Boothe and Boothe 2015). For this purpose, administration of the first-generation cephalosporin is recommended before surgery planned to last for one hour (Valkki et al. 2020). While some studies have reported that antimicrobial prophylaxis reduces the incidence of SSIs (Eugster et al. 2004), other studies have indicated that prophylactic antimicrobial therapy is ineffective in reducing infection rates (Spencer and Daye 2018; Stetter et al. 2021).

Pet animals, especially cats and dogs, nowadays interact with humans intimately as never before. The resistant bacteria, which they carry in their body, pose a significant risk to public health (Walther et al. 2012). Moreover, these agents can lead to failure and higher costs in treatment (Awosile et al. 2018). Therefore, monitoring the antimicrobial resistance profiles of bacteria isolated from pet animals with up-to-date data is crucial to reveal the potential risk to public health and determine effective treatment protocols.

Studies have reported that superficial or deep wound infections are more common in companion animals after surgeries (42.0–81.3% and 6.3–50.0%, respectively) than organ/space infections (Turk et al. 2015; Espinel Ruperez et al. 2019). Especially in surgeries involving implant applications, it is emphasised that bacterial agents able to form biofilms play a role in resistance development and become colonised in the surgical site (Khatoon et al. 2018). Cephalosporin group antibiotics and marbofloxacin are suggested for prophylactic purposes (Ferran et al. 2016; Valkki et al. 2020). However, the fact that antimicrobial resistance has become a global problem is increasingly emphasising the importance of effective antibiotic use in veterinary medicine.

Despite an increase in the asepsis-antisepsis, disinfection, and other practices (e.g. considering daily disinfection of hospitalisation units and basic biosecurity practices, hospitalising animals with infectious diseases in quarantine units, etc.) at Siirt University Animal Health Application and Research Hospital, the occurrence of post-orthopaedic surgery infections was not exactly eliminated. The prolonged treatment processes and negative impacts on prognosis have led researchers to investigate the antimicrobial resistance profiles of bacterial agents causing these infections and determine effective treatment options.

Therefore, this study aims to identify the bacteria causing SSIs in cats and dogs following orthopaedic surgeries, including mostly implant applications, and determine their antimicrobial resistance profiles.

## MATERIAL AND METHOD

### Ethical considerations

The Siirt University Animal Experiments Local Ethics Committee (Approval No.: 2024/01/01) approved this study.

### Animals

The sample under study consisted of 21 cats and 19 dogs brought to the Surgical Clinic of Siirt University Animal Health Application and Research Hospital diagnosed with extremity fractures based on clinical and radiological examinations. Among the cats, 16 were hybrid breeds, 3 were Van Cat, and 2 were Scottish Fold. Their ages ranged from 3 months to 4 years, and their weights ranged between 1.0 and 4.6 kg. On the other hand, 18 dogs were hybrid breeds and one was a Belgian Malinois. The ages of the dogs ranged from 2 months to 11 years and their weights ranged between 6 and 34 kg.

### Preoperative care

The subjects diagnosed with extremity fractures after routine clinical and radiological examinations were operated within 1 to 3 days. Prophylactic antibiotic administration was initiated one hour before the surgery and continued until signs of infection at the suture line were observed.

### Operative techniques and postoperative care

The subjects were fasted for 12 h before the operation. After shaving and disinfecting the fracture site, induction was performed with 2 mg/kg intramuscular xylazine HCL (Alfazyne^®^ 2%; Egevet, Türkiye) and 10 mg/kg intramuscular ketamine HCL (Alfamine^®^ 10%; Egevet, Türkiye). Subsequently, the patients were intubated and connected to a closed-circuit anaesthesia machine (SMS 2000 Classic Vent-V, SMS Medical Device; Elec. Elekt. Const. Tex. Turz. Oto Ind. and Tic. Ltd. Şti., Ankara, Türkiye). Anaesthesia maintenance was achieved with 2% sevoflurane (Sevorane^®^; Abbott, Italy). Routine asepsis-antisepsis procedures (chlorhexidine, isopropyl alcohol) were applied, and the surgical method was selected based on the classification of the fractures. During the postoperative period, all patients’ surgical wounds were examined daily. A swab sample was taken for microbiological analysis upon clinical findings such as redness, temperature increase, oedema, and purulent and malodorous discharge in the relevant area. Thus, a sterile cotton-tipped swab was applied to the wound area by rotating the wound line following the asepsis-antisepsis rules. It was placed in tubes containing a sterile transport medium (Gülka Kimya, Ankara, Türkiye) and sent to the microbiology laboratory for bacterial isolation and identification.

### Isolation and identification

Swab samples were cultured on blood agar containing 5% defibrinated sheep blood (Oxoid, CM0271, England) and incubated aerobically at 37 °C for 24–48 hours. After the incubation period, pure cultures were obtained from colonies grown in the culture medium. The Gram staining, microscopic morphology, catalase, oxidase and coagulase reactions of the colonies were evaluated. For the identification of the isolates at the genus and species levels, they were sent to Hatay University Plant Health Clinic Application and Research Centre. Protein fingerprints were obtained from isolates by the ethanol formic acid extraction method using the matrix-assisted laser desorption ionization-time of flight mass spectrometry (MALDI-TOF MS). Microorganisms were identified by library scanning as a spectrum match. The spectra obtained with the device’s flex control software programme (Biotyper 3.0; Microflex LT; Bruker Daltonics GmbH, Bremen, Germany) were compared with maldi biotyper real-time classification (RTC) software (v9), and the identification process was performed (Uysal et al. 2019).

### Determination of antimicrobial susceptibility

The disc diffusion method was used to determine the antimicrobial susceptibility of the isolates. Antibiotics were selected according to the bacteria species in line with the criteria reported by CLSI (2018) and EUCAST (2019).

For *Staphylococcus* spp., gentamicin (10 μg, Liofilchem), rifampicin (5 μg, Liofilchem), penicillin G (10 IU, Liofilchem), cefoxitin (30 μg, Liofilchem), cefpodoxime (10 μg, Liofilchem), enrofloxacin (5 μg, Liofilchem), sulphamethoxazole + trimethoprim (25 μg, Liofilchem), clindamycin (2 μg, Liofilchem), erythromycin (15 μg, Liofilchem), chloramphenicol (30 μg, Liofilchem), tetracycline (30 μg, Liofilchem), ciprofloxacin (5 μg, Liofilchem), and doxycycline (30 μg, Liofilchem) disks were used.

For *Streptococcus* spp., cefpodoxime (10 μg, Liofilchem), enrofloxacin (5 μg, Liofilchem), clindamycin (2 μg, Liofilchem), tetracycline (30 μg, Liofilchem), and chloramphenicol (30 μg, Liofilchem) disks were used.

For *Enterococcus* spp., ampicillin (10 μg, Lio-filchem), penicillin G (10 IU, Liofilchem), erythromycin (15 μg, Liofilchem), chloramphenicol (30 μg, Liofilchem), tetracycline (30 μg, Liofilchem), imipenem (10 μg, Liofilchem), ciprofloxacin (5 μg, Liofilchem), and vancomycin (5 μg, Liofilchem) disks were used.

For *Enterobacteriaceae*, gentamicin (10 μg, Liofilchem), streptomycin (10 μg, Liofilchem), enrofloxacin (5 μg, Liofilchem), ciprofloxacin (5 μg, Liofilchem), tetracycline (30 μg, Liofilchem), sulfamethoxazole + trimethoprim (25 μg, Liofilchem), piperacillin + tazobactam (100/10 μg, Liofilchem), chloramphenicol (30 μg, Liofilchem), imipenem (10 μg, Liofilchem) and ertapenem (10 μg, Liofilchem) disks were used.

For *Pseudomonas* spp., gentamicin (10 μg, Lio-filchem), piperacillin + tazobactam (100/10 μg, Liofilchem), imipenem (10 μg, Liofilchem), enrofloxacin (5 μg, Liofilchem), aztreonam (30 μg, Liofilchem), and ciprofloxacin (5 μg, Liofilchem) disks were used.

Cefoxitin (30 μg, Liofilchem) disk was considered for methicillin resistance in *Staphylococcus* spp. isolates. For the identification of high-level aminoglycoside resistance (HLAR) and high-level streptomycin resistance (HLSR) in enterococci, gentamicin (30 μg, Oxoid) and streptomycin (300 μg, Oxoid) disks were used, respectively (EUCAST 2019). To determine the extended spectrum of beta lactamase (ESBL) resistance in *Enterobacteriaceae* isolates, a combined disk method was used (CLSI 2018). The combined disk method reported by Kaplan and Gulaydin (2023) was used to identify the plasmid-mediated AmpC beta-lactamase resistance in isolates. Test results were evaluated as susceptible, intermediate, and resistant based on the specified criteria. Accordingly, resistance to at least one antimicrobial substance among antimicrobials belonging to three or more categories was considered multidrug resistance (Magiorakos et al. 2012).

## RESULTS AND DISCUSSION

Table 1 shows the clinical diagnosis and the operation method used.

**Table 1:** Information regarding the clinical diagnosis and the applied surgical method in patients under study

Case No.	Species	Clinical diagnosis	Surgical method
1	cat	right radius-ulna proximal open infected fracture	external fixation
2	cat	left femur midshaft closed fracture	im pin osteosynthesis
3	cat	bilateral femur supracondylar closed fracture	cp osteosynthesis
4	cat	right femur supracondylar open fracture	cp osteosynthesis
5	cat	left femur supracondylar closed fracture	cp osteosynthesis
6	cat	right femur supracondylar closed fracture	cp osteosynthesis
7	cat	left tibia distal diaphyseal open infected fracture	external fixation
8	cat	right humerus open infected fracture with tissue loss	amputation
9	cat	left tibia midshaft closed fracture	im pin osteosynthesis
10	cat	left femur distal diaphyseal open fracture	im pin osteosynthesis
11	cat	right humerus distal diaphyseal open infected fracture	external fixation
12	cat	bilateral tibia distal diaphyseal open infected fracture	external fixation
13	cat	right humerus distal diaphyseal closed fracture	im pin osteosynthesis
14	cat	left humerus midshaft closed fracture	external fixation
15	cat	right calcaneus proximal closed fracture	cp osteosynthesis
16	cat	left humerus midshaft open infected fracture with tissue loss	amputation
17	cat	right humerus proximal diaphyseal closed fracture	im pin osteosynthesis
18	cat	left metatarsus extensive loss open infected fracture	amputation
19	cat	right femur supracondylar fracture	cp osteosynthesis
20	cat	right femur supracondylar fracture	cp osteosynthesis
21	cat	left femur supracondylar fracture	cp osteosynthesis
22	dog	right tibia midshaft open fracture	external fixation
23	dog	left radius-ulna diaphyseal closed fracture	im pin osteosynthesis
24	dog	right radius-ulna diaphyseal closed fracture	im pin osteosynthesis
25	dog	left radius-ulna diaphyseal open fracture	im pin osteosynthesis
26	dog	left radius-ulna proximal open infected fracture with tissue loss	amputation
27	dog	left femur supracondylar closed fracture	cp osteosynthesis
28	dog	left radius-ulna extensive tissue loss open infected fracture	amputation
29	dog	right tibia midshaft closed fracture	im pin osteosynthesis
30	dog	left femur midshaft closed fracture	im pin osteosynthesis
31	dog	left tibia distal diaphyseal closed fracture	im pin osteosynthesis
32	dog	right tibia proximal transverse fracture	im pin osteosynthesis
33	dog	left femur distal diaphyseal closed fracture	im pin osteosynthesis
34	dog	left tibia midshaft closed fracture	plate osteosynthesis
35	dog	right femur distal diaphyseal open fracture	external fixation
36	dog	left metatarsus open infected fracture with tissue loss	amputation
37	dog	left radius-ulna diaphyseal closed fracture	im pin osteosynthesis
38	dog	right radius-ulna diaphyseal closed fracture	im pin osteosynthesis
39	dog	left tibia distal diaphyseal closed fracture	external fixation
40	dog	right femur midshaft closed fracture	im pin + external fixation

cp = cross pin; im pin = intramedullary pin

Pure cultures were obtained from 32 swab samples (80%), while more than one bacteria species were isolated from 8 samples (20%). The highest isolation rate was determined in coagulase-positive *Staphylococcus* spp. (CPS) (29.78%) isolates [*Staphylococcus pseudintermedius* (*S.* *pseudintermedius*) *n* = 13, *Staphylococcus aureus* (*S. aureus*) *n* = 1], followed by *Escherichia coli* (*E. coli*) (17.02%), *Enterobacter cloacae* (*E. cloacae*) (10.63%), *Klebsiella pneumoniae* (*K. pneumoniae*) (10.63%), and *Enterococcus faecalis* (*E. faecalis*) (6.38%) (Table 2).

**Table 2:** Distribution of isolated bacteria in the study

Bacteria	*n*	%
*S. pseudintermedius*	10	21.27
*E. coli*	6	12.76
*K. pneumoniae*	4	8.5
*P. mirabilis*	3	6.38
*E. cloacae*	3	6.38
*S. canis*	2	4.25
*E. faecalis*	2	4.25
*E. faecium*	1	2.12
*S. haemolyticus*	1	2.12
*S. aureus*	1	2.12
*S. marcescens*	1	2.12
*P. aeruginosa*	1	2.12
*E. faecalis* + *E. cloacae*	1	2.12
*S. pseudintermedius* + *E. cloacae*	1	2.12
*E. faecium* + *P. aeruginosa*	1	2.12
*S. pseudintermedius* + *E. coli*	1	2.12
*S. pseudintermedius* + *P. aeruginosa*	1	2.12
*K. pneumoniae* + *E. cloacae*	1	2.12

Table 3 shows the distribution of the antimicrobial susceptibility of the isolates. Among the 14 CPS isolates, 78.57% had methicillin resistance. Methicillin resistance was determined in 76.92% of *S. pseudintermedius* isolates (MRSP). All MRSP isolates had multidrug resistance. *S. aureus* isolate was resistant to methicillin (MRSA) and did not exhibit multidrug resistance. While HLAR was identified in 60% of enterococcal strains, HLSR was found in 20% of them. Additionally, 18.18% of *Enterobacteriaceae* isolates were determined as EBSL producers while 13.63% of them had AmpC resistance. Furthermore, 40.90% of the isolates exhibited ESBL and plasmidic AmpC resistance. Accordingly, the total ESBL resistance rate among pathogens classified within the *Enterobacteriaceae* family was 59.09%, and the total plasmid AmpC resistance rate was 54.54% (Table 4).

**Table 3:** Distribution of antimicrobial susceptibility of isolates obtained in the study

Antimicrobials	*Staphylococcus* spp. (*n* = 15)				*Streptococcus* spp. (*n* = 2)				*Enterococcus* spp. (*n* = 5)				*Enterobacteriaceae* (*n* = 22)				*P. aeruginosa* (*n* = 3)		
	S	I	R		S	I	R		S	I	R		S	I	R		S	I	R
Gentamicin	9	1	5		–	–	–		–	–	–		20	1	1		2	0	1
Rifampicin	4	0	11		–	–	–		–	–	–		–	–	–		–	–	–
Penicillin	3	0	12		–	–	–		3	0	2		–	–	–		–	–	–
Cefoxitin	3	0	12		–	–	–		–	–	–		–	–	–		–	–	–
Cefpodoxime	3	0	12		2	0	0		–	–	–		–	–	–		–	–	–
Enrofloxacin	4	1	10		2	0	0		–	–	–		11	3	8		2	0	1
Sulphamethoxazole + trimethoprim	4	1	10		–	–	–		–	–	–		11	0	11		–	–	–
Clindamycin	4	1	11		2	0	0		–	–	–		–	–	–		–	–	–
Erythromycin	4	0	11		–	–	–		1	0	4		–	–	–		–	–	–
Chloramphenicol	14	0	1		2	0	0		4	0	1		18	0	4		–	–	–
Tetracycline	2	4	9		0	0	2		2	2	1		4	2	16		–	–	–
Ciprofloxacin	3	0	11		–	–	–		1	0	4		13	0	9		2	0	1
Doxycycline	5	1	9		–	–	–		–	–	–		–	–	–		–	–	–
Streptomycin	–	–	–		–	–	–		–	–	–		13	5	4		–	–	–
Piperacillin + tazobactam	–	–	–		–	–	–		–	–	–		15	2	5		3	0	0
Imipenem	–	–	–		–	–	–		1	0	4		8	2	12		1	0	2
Ertapenem	–	–	–		–	–	–		–	–	–		16	0	6		–	–	–
Vancomycin	–	–	–		–	–	–		5	0	0		–	–	–		–	–	–
Ampicillin	–	–	–		–	–	–		3	0	2		–	–	–		–	–	–
Aztreonam	–	–	–		–	–	–		–	–	–		–	–	–		3	0	0

(–) = antimicrobials were selected according to the bacteria species, therefore, some results were missing for some species; I = intermediate; R = resistant; S = susceptible

**Table 4:** Resistance profile of the strains isolated from the cases and treatment protocol applied for prophylactic purposes and after antibiogram

No.	Prophylactic antibiotic treatment	Isolated bacteria	Resistance profile	Revised antibiotic treatment	Infection healing time (d)
1	cefazolin sodium, 4 days, 25 mg/kg	*S. pseudintermedius*	TE(I)	enrofloxacin, 7 days, 5 mg/kg	3
2	ceftriaxone, 7 days, 25 mg/kg	*E. faecalis*	CIP+E+TE(I)+**HLAR**	clindamycin, 10 days, 10 mg/kg	4
		*E. cloacae^**†**^*	GEN(I)+ENR+CIP+TE+SXT+TPZ+IMP+ETP+**ESBL+AmpC**		
3	cefuroxime, 5 days, 20 mg/kg	S. pseudintermedius*	RD+P+**FOX**+CPD+ENR+SXT+DA+E+TE(I)+CIP+DXT	gentamicin, 5 days, 4 mg/kg	2
		*E. cloacae^**†**^*	S(I)+ENR+CIP+TE+SXT+TPZ+IMP+ETP+**ESBL+AmpC**		
4	ceftriaxone, 5 days, 25 mg/kg	*E. coli*	TE(I)+IMP(I)+**AmpC**	enrofloxacin, 7 days, 5 mg/kg	2
5	enrofloxacin, 4 days, 5 mg/kg	*S. canis*	TE	clindamycin, 7 days, 10 mg/kg	4
6	enrofloxacin, 3 days, 5 mg/kg	S. pseudintermedius*	RD+P+**FOX**+CPD+ENR+SXT+DA+E+TE+CIP+DXT	gentamicin, 5 days, 4 mg/kg	2
7	ceftriaxone, 3 days, 25 mg/kg	*S. canis*	TE	enrofloxacin, 7 days, 5 mg/kg	4
8	cefazolin, 7 days, 25 mg/kg	*E. faecalis*	CIP+C+P+E+IMP	enrofloxacin, 7 days, 5 mg/kg	3
9	ceftriaxone, 7 days, 25 mg/kg	*E. faecium*	CIP+P+E+AMP+IMP+TE(I)+**HLSR**	vancomycin, 5 days, 15 mg/kg	2
10	enrofloxacin, 5 days, 5 mg/kg	*P. mirabilis*	ENR(I)+TE+SXT	gentamicin, 5 days, 4 mg/kg	2
11	cefazolin, 4 days, 25 mg/kg	*K. pneumoniae^**†**^*	S+TE(I)+SXT+TPZ+IMP+ETP+**ESBL**	enrofloxacin, 7 days, 5 mg/kg	3
12	cefuroxime sodium, 3 days, 20 mg/kg	S. pseudintermedius*	RD+P+**FOX**+CPD+ENR(I)+SXT+DA+E+TE(I)CIP+DXT(I)	gentamicin, 5 days, 4 mg/kg	2
13	ceftriaxone, 7 days, 25 mg/kg	*P. mirabilis^**†**^*	TE+SXT+IMP+**ESBL**+**AmpC**	pipercillin-tazobactam, 7 days, 50 mg/kg	2
14	cefazolin sodium, 7 days, 25 mg/kg	*S. haemolyticus*	GEN+RD+P+**FOX**+CPD+ENR+SXT+DA+E+CHL+TE+CIP	doxycycline, 5 days, 10 mg/kg	3
15	enrofloxacin, 4 days, 5 mg/kg	*E. cloacae*	ENR+CIP+TE+SXT+C+IMP+**AmpC**	piperacillin + tazobactam, 7 days, 50 mg/kg	3
16	cefazolin sodium, 7 days, 25 mg/kg	*P. mirabilis^**†**^*	S(I)+ENR(I)+TE+SXT+IMP+**ESBL**+**AmpC**	piperacillin + tazobactam, 7 days, 50 mg/kg	2
17	cefazolin sodium, 7 days, 25 mg/kg	*K. pneumoniae^**†**^*	S+ENR(I)+CIP+TE+SXT+TPZ(I)+IMP+ETP+**ESBL**	enrofloxacin, 7 days, 5 mg/kg	3
18	cefazolin sodium, 7 days, 25 mg/kg	S. aureus**	P+**FOX**+CPD	sulfamethoxazole + trimethoprim, 7 days, 16 mg/kg	3
19	cefazolin sodium, 6 days, 25 mg/kg	S. pseudintermedius*	GEN+RD+P+**FOX**+CPD+ENR+SXT+DA+E+TE+CIP+DXT	sulfamethoxazole + trimethoprim, 7 days, 16 mg/kg	4
		*E. coli^**†**^*	S(I)+TE+TPZ+IMP+ETP+**ESBL**+**AmpC**		
20	enrofloxacin, 5 days, 5 mg/kg	*E. coli^**†**^*	S(I)+ENR+ CIP+TE+TPZ(I)+C+IMP(I)+**ESBL**	sulfamethoxazole + trimethoprim, 7 days, 16 mg/kg	2
21	cefazolin sodium, 6 days, 25 mg/kg	*E. faecium*	AMP+IMP+TE+GEN+**HLAR**	piperacillin + tazobactam, 7 days, 50 mg/kg	3
		*P. aeruginosa*	GEN+IMP+ENR+CIP		
22	cefazolin sodium, 4 days, 25 mg/kg	*E. coli^**†**^*	S+ENR+CIP+TE+SXT+C+**ESBL**	gentamicin, 7 days, 4 mg/kg	3
23	cefazolin sodium, 5 days, 25 mg/kg	*K. pneumoniae^**†**^*	**ESBL**+**AmpC**	enrofloxacin, 7 days, 5 mg/kg	3
24	cefazolin sodium, 5 days, 25 mg/kg	*E. coli*	–;	piperacillin + tazobactam, 7 days, 50 mg/kg	2
25	cefazolin sodium, 7 days, 25 mg/kg	*E. coli*	TE(I)+IMP(I)+**AmpC**	enrofloxacin, 7 days, 5 mg/kg	2
26	cefazolin sodium, 5 days, 25 mg/kg	*S. pseudintermedius*	TE(I)	gentamicin, 5 days, 4 mg/kg	2
27	cefazolin sodium, 5 days, 25 mg/kg	*S. marcescens*	TE+IMP	ciprofloxacin, 7 days, 20 mg/kg	2
28	ceftriaxone, 7 days, 25 mg/kg	S. pseudintermedius*	RD+P+**FOX**+CPD+ENR+SXT+DA+E+TE+CIP+DXT	gentamicin, 7 days, 4 mg/kg	3
29	ceftriaxone, 7 days, 25 mg/kg	*P. aeruginosa*	–	piperacillin + tazobactam, 7 days, 50 mg/kg	2
30	enrofloxacin, 7 days, 5 mg/kg	*E. faecalis*	CIP+E+IMP(I)+GEN+**HLAR**	cefoxitin, 7 days, 20 mg/kg	3
31	cefazolin sodium, 5 days, 25 mg/kg	*E. cloacae*	–	enrofloxacin, 7 days, 5 mg/kg	3
32	cefazolin sodium, 7 days, 25 mg/kg	*S. pseudintermedius*	–	enrofloxacin, 7 days, 5 mg/kg	4
33	ampicillin sulbactam, 3 days, 20 mg/kg	S. pseudintermedius*	RD+P+**FOX**+CPD+ENR+SXT+DA+E+TE+CIP+DXT	gentamicin, 5 days, 4 mg/kg	3
		*E. coli^**†**^*	ENR+CIP+TE+SXT+C+**ESBL**		
34	cephalexin, 7 days, 15 mg/kg	S. pseudintermedius*	GEN(I)+RD+P+**FOX**+CPD+ENR+SXT+DA+E+TE+CIP+DXT	gentamicin, 5 days, 4 mg/kg + chloramphenicol (pomade)	3
35	cephalexin, 7 days, 15 mg/kg	S. pseudintermedius*	GEN+RD+P+**FOX**+CPD+ENR+SXT+DA+E+TE+CIP+DXT	chloramphenicol (pomade)	4
36	ceftriaxone, 7 days, 25 mg/kg	*E. coli*	S+ENR+CIP+TE+IMP+SXT	piperacillin + tazobactam, 7 days, 50 mg/kg	3
37	ceftriaxone, 6 days, 25 mg/kg	S. pseudintermedius*	GEN+RD+P+**FOX**+CPD+ENR+SXT(I)+DA+E+TE+CIP+DXT	ciprofloxacin, 7 days, 20 mg/kg + chloramphenicol (pomade)	2
		*P. aeruginosa*	IMP		
38	cefazolin sodium, 7 days, 25 mg/kg	*K. pneumoniae^**†**^*	TE+**ESBL**+**AmpC**	piperacillin + tazobactam, 7 days, 50 mg/kg	2
		*E. cloacae*	TE+**ESBL**+**AmpC**		
39	cefazolin sodium, 9 days, 25 mg/kg	S. pseudintermedius*	GEN+RD+P+**FOX**+CPD+ENR+SXT+DA+E+TE+CIP+DXT	chloramphenicol (pomade)	4
40	cefazolin sodium, 5 days, 25 mg/kg	*E. cloacae^**†**^*	GEN+ENR+CIP+TE+SXT+TPZ+IMP+ETP+**ESBL**+**AmpC**	chloramphenicol (pomade)	4

(I) = intermediate; * = multidrug-resistant MRSP; ** = methicillin-resistant *Staphylococcus aureus*; † = ESBL-resistant *Enterobacteriaceae*; AMP = ampicillin; AmpC = plasmid mediated AmpC beta lactamase; C = chloramphenicol; CIP = ciprofloxacin; CPD = cefpodoxime; DA = clindamycin; DXT = doxycycline; E = erythromycin; ENR = enrofloxacin; ESBL= extended-spectrum beta-lactamase; ETP = ertapenem; FOX = cefoxitin; GEN = gentamicin; IMP = imipenem; P = penicillin; RD = rifampin; S = streptomycin; SXT = sulfamethoxazole + trimethoprim; TE = tetracycline; TPZ = piperacillin + tazobactam; VA = vancomycin

While 85% of the 40 cases obtained the prophylactic treatment with beta-lactam/cephalosporin group antimicrobial agents before the operation, in the postoperative period cephalosporin treatment was administered only to one patient (case No. 30) based on the antimicrobial susceptibility results. Table 4 shows the antibiotic treatment protocols applied in the pre- and post-operative periods, along with the isolated bacteria and resistance profiles.

SSIs have a multifactorial aetiology and may occur despite meticulous postoperative precautions (Turk et al. 2015; Windahl et al. 2015). Numerous studies have reported that this complication increases morbidity and mortality and may lead to prolonged hospitalization, increased treatment costs, and negative impacts such as emotional pressure on the pet owner and the veterinarian (Eugster et al. 2004; Turk et al. 2015). Particularly, the difficulty in antimicrobial treatment of infections caused by bacterial agents with multiple antibiotic resistances poses a significant concern for veterinarians (Turk et al. 2015).

When examining previous studies on the subject, it was found that the overall infection rate after minor surgical operations generally ranges between 3% and 10% (Beal et al. 2000; Nicholson et al. 2002; Eugster et al. 2004; Fitzpatrick and Solano 2010; Frey et al. 2010; Turk et al. 2015). In the present study, the number of operations performed and the records of SSI development during the study period were not documented because there was no patient tracking system at the time the study was conducted. Also, the study was designed based on microbiological evaluation in cases where SSI symptoms were clinically observed, and the SSI incidence rate could not be calculated.

Costerton (2005) revealed that the rate of SSIs was 5 to 6 times higher in operations using implants than in those without implants. This situation can be explained by the possibility of surgical implants becoming contaminated with bacteria, and the potential of implant surfaces to serve as substrates for biofilm formation of bacteria (Turk et al. 2015). In the present study, SSIs were determined after orthopaedic surgeries with implants used in 34 (85%) of 40 cases.

Numerous studies have been conducted to determine the prevalence of bacteria in SSIs. These studies have generally emphasised that *Staphylococcus* spp. is the most commonly isolated pathogen (Sen and Kilic 2012; Windahl et al. 2015; Gomez Beltran et al. 2020; Nocera et al. 2021; Karakaya Bilen et al. 2023). Also, the majority of these isolates were identified as *S. pseudintermedius* (Windahl et al. 2015; Gomez Beltran et al. 2020; Nocera et al. 2021). Consistent with other studies, *Staphylococcus* spp. was the most frequently isolated one in this study. The majority of the isolates were identified as *S. pseudintermedius*.

In recent studies, the isolation rate of methicillin-resistant CNS (MRCNS) from various clinical samples of cats and dogs has been reported to range from 3% to 25% (Gocmen et al. 2020; Elnageh et al. 2021; Gulaydin et al. 2022). In the presented study, MRCNS (*Staphylococcus haemolyticus*) was isolated in one case (2.5%) and was resistant to methicillin. On the other hand, Karakaya Bilen et al. (2023) reported a higher rate of MRCNS isolates (45%) compared to other studies.

Turk et al. (2015) mentioned in their studies that MRSP isolates were the most common cause of SSIs. Valkki et al. (2020) reported that the isolation rate of MRSP was 23.07%. Windahl et al. (2015) indicated a high level of multidrug resistance in *Staphylococcus* spp. They also found that 26% of *S. pseudintermedius* isolates were multidrug resistant. Similarly, in this study, methicillin and penicillin resistance were detected in 80% of *Staphylococcus* spp. isolates, and high methicillin resistance was found in *S. pseudintermedius* isolates. In contrast to Windahl et al. (2015), in this study, it was found that all MRSP isolates (100%) had multidrug resistance.

ESBL-producing *Enterobacteriaceae* strains have zoonotic potential (Windahl et al. 2015). In several studies, ESBL-producing *E. coli* strains were isolated from pets (O’Keefe et al. 2010; Smet et al. 2010; Windahl et al. 2015; Kaplan and Gulaydin 2023). In the present study, ESBL resistance was high in *Enterobacteriaceae* strains, and it was concluded that this situation would adversely affect both public health and the health of cats and dogs. Additionally, the isolation of ESKAPE pathogens (*E. faecium*, *S. aureus*, *K. pneumoniae*, *P. aeruginosa*, and *Enterobacter* spp.) in this study suggested that the infections had a nosocomial nature (Santaniello et al. 2020).

Cephalosporin group antibiotics are preferred for prophylactic purposes due to their rapid passage between plasma and the surgical wound, broad-spectrum, high tissue concentrations, low toxicity, and low cost (Valkki et al. 2020).

However, the development of resistance to these antibiotics in bacteria (Gulaydin et al. 2022; Kaplan and Gulaydin 2023) limits their use. In this study, in only one of 40 patients, cephalosporin was used postoperatively. Additionally, beta lactam/cephalosporin resistant strains were isolated from 39 SSI cases.

In this study, MRSP and ESBL-producing *Ente-robacteriaceae* strains emerged as the predominant agents of SSIs in cat and dog orthopaedic surgeries. The findings underscored the potential risks associated with prophylactic antibiotic use, which may contribute to the development of resistance in bacteria.

The importance of determining the aetiology and resistance profile of bacteria in SSI was highlighted. These measures are deemed crucial for effectively treating SSIs and preventing the development of multidrug-resistance in bacteria.
